# The Roots of Bioinformatics in ISMB

**DOI:** 10.1371/journal.pcbi.1002679

**Published:** 2012-08-30

**Authors:** Todd A. Gibson

**Affiliations:** Computer Science Department, California State University, Chico, California, United States of America

In 1988, Disney released *Oliver & Company*, their first animated movie to feature widespread use of computer-generated imagery (CGI). The use of CGI in films was entering the mainstream of Hollywood, and it marked one of the few places at the time that had a demand for artificial intelligence (AI) researchers and the large computing facilities to support them. Lawrence (Larry) Hunter had just graduated with a PhD in artificial intelligence from Yale, and was familiar with Hollywood, having grown up there. But rather than pursuing an AI career in cinema, Hunter went on to gather a small group of like-minded scientists, and together they established what has become the largest international conference in computational biology: Intelligent Systems for Molecular Biology (ISMB). Since its inception, ISMB has held meetings on four continents and published proceedings papers from researchers in 34 different countries. This year, ISMB celebrated its 20th annual international conference in Long Beach, California. Through interviews with many who played prominent roles in the formation of ISMB, we look back at the early days of the field from which ISMB was born. Their reflections provide insight into the early meetings, and the growth and maturation of both ISMB and the field it represents over the last two decades.

## Before ISMB, the Early Years

In the fall of 1988, Lawrence Hunter completed his doctoral dissertation, which included case-based reasoning to diagnose lung tumors from histological slides. It's precisely what you would expect for someone enthusiastically pursuing the intersection of biology and computation. Only Hunter wasn't enthusiastic:

I was no longer afraid of walking around in a hospital, I was no longer terrified of being mistaken for a patient. I got used to looking at gross photographs of bits and pieces of people (they don't call it gross anatomy for nothing!), and I'd learned a certain amount of medical terminology. I wasn't motivated by medicine in those days, I wanted to stop doing lung tumor pathology pretty badly. Lung tumor pathology, especially in the '80s, there really wasn't very much we could do. If you had lung cancer, you were pretty much out of luck. Everybody died in six months-ish…. I spent all this time trying to get a computer program to make these distinctions that had absolutely no clinical significance whatsoever. So that was really depressing, the fact that everybody died, and the therapies were miserable to undergo and didn't really help very much…

I was pretty systematic, starting in around the middle of 1988, trying to figure out where I was going to get a job that wasn't going to make me feel miserable or how I was going to make a living.

Hollywood was a natural fit for Hunter's skills. Computer animation was taking off and there was a strong demand for AI skills which could reduce the human labor required to animate a film. However, despite having grown up there (or perhaps because of it), Hunter avoided it:

I knew what the film business was like, and I knew I did not want to do that…. It sounded sexy except that no job lasted more than 6 months and it's a constant scramble. I had a lot of friends who wanted to go into the film business. I knew what kind of suffering those kids went through, how miserable it was to try and get those jobs, how brutal the competition was and how unfair.

At the same time, the United States National Institutes of Health (NIH) was in a political battle with the Department of Energy (DoE) over the Human Genome Project (which began in October of 1990). Though today nobody would question the province of NIH to control the Human Genome Project, at the time GenBank was run by the DoE's Los Alamos National Laboratory, and the DoE had experience breaking up genomes using radiation hybrids. The NIH sought to strengthen its argument to control the Human Genome Project by establishing its computer and engineering skills. In March 1989, Hunter was one of the first, if not the first, computer scientist hired as a staff scientist at NIH's National Library of Medicine (NLM).

## Planning: The 1991 Pre-ISMB Workshop

At NLM Hunter maintained a database of AI researchers interested in molecular biology, and with it the groundwork was laid for ISMB in November, 1991:

I got a little money from both [NIH and NSF] to invite people in for a two-day workshop. The invitations were based on the database. That was the meeting that created the first ISMB. We decided at that meeting that we needed a journal, a conference, a summer school, and a scientific society. That's what a “real field” has…. It was a really interesting meeting, it felt really heady. It felt like we were really onto something.

David Searls also recalls the workshop:

You have to understand how new this was at that time. There really wasn't much there so they sponsored…a workshop to try to bootstrap infrastructure in this field.

It's worth remembering that there were people working on computational applications in structural biology…and also phylogenetics…. The algorithms community, computer science community, had started to get involved, mainly with string algorithms.

What was really missing from all that was the AI attitude, which is basically symbolic processing and associated things like machine learning and databases, and so forth.

[The November 1991 workshop] group was mainly interested in bootstrapping an approach to computational biology that was more grounded in AI. At the same time, I don't think we wanted to tie ourselves to AI because the purpose of AI is machine intelligence, and our interest was in aiding biology, or the application of computers to biology. We didn't want to put the focus on intelligence, but rather on the methodologies that we used in AI…. I think Larry felt that it was time to establish a meeting that would serve as the touchstone for this whole approach that we were thinking about.

## The Early Meetings

A tremendous amount of effort goes into organizing and running the ISMB meeting each year. But whereas recent ISMB meetings benefit from the experience and organization that has built up over the many years of running the meeting, the early meetings had no such scaffolding to rely on. In July 1993, the first ISMB meeting was held where Hunter worked, at the Lister Hill Center, which was the basic research arm of the NLM. Searls recalls:

We all wrote a grant…to run the meeting. It wasn't much. We did it on a shoestring…. The program covers one 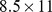 sheet, both sides, which I folded into three and made a nice, neat schedule, complete with the poster session. I kind of remember, now, putting that together on my early Mac. We did things pretty much on the cheap. We didn't have meeting organizers.

There were people milling around in the lobby [of the hotel]. I sat down at a table, and just started registering people. We went to the Lister Hill Center, the auditorium there, to actually conduct the meetings. At that time, I think we could just hike across the field into the NIH grounds. Nowadays, it's a walled fortress so you can't get in, but in those days, we just hiked over to the Lister Hill Center, to the auditorium there, and ran the meeting.

Jude Shavlik recalls the ad hoc feel as well:

We were turning people away because there wasn't enough room. We spent a lot of trouble [planning for] an overflow room…. It was pretty high demand right away…. Of course, some people didn't want to come because we said that late registrants could only go to the overflow room. By the time the conference really happened, maybe the first morning, people had to go in the overflow room. Then after that, with people hanging out in the halls or leaving early or whatnot, we were fine using the regular room. But it was an immediate sell out, at least at a couple hundred.

Searls remembers some elation after the first ISMB:

It was very exciting. It was hard work…. I think we were exhausted. I remember after the meeting, Larry and Jude and I decided to go out to lunch to unwind and sort of bask in the glory.

We went to a place in Bethesda, a local place that Larry knew. I think, first of all, we breathed a sigh of relief to have it over with, but…I think we were very excited that this meeting had been successful and it showed promise of continuing and growing. There was a sense, even at that point, of a birth having occurred and that something good was happening.

There's an emotional element to it at the time. It was exciting to have this happening at the same time that the idea of the genome project was now on the horizon.

There was a sense that the data was just going to keep on coming, and the challenges were going to keep on coming, along with new technologies, and that we had the computational tools to grow in tandem with the demand….

We called it the “First Annual” meeting, and we expected it to be annual from then on. I was pretty optimistic at that time that that was the expectation. We wanted to have the signature meeting for this community of computational biologists.

All of the early ISMB meetings were challenging to prepare and run. Hunter finds the second ISMB particularly memorable:

I think the most memorable was 1994, the second ISMB meeting. We had planned to hold it in Seattle…. We announced the Seattle ISMB at the conclusion of the first meeting in July '93. Then, by November of '93, we discovered [that the Seattle organizer] was organizing a competing meeting just a month before when ISMB '94 was to be, and he had basically stopped talking to us. In a panic, we cast about for some other location where we could hold the meeting with only about 6 months lead time to get organized.

Russ Altman picks up from here:

I got a pretty late call from Larry saying, “Russ, we don't have a venue for the next meeting and it's not going to happen unless we put it together.” And I said, “Well, why don't we try to have it at Stanford? I can get an auditorium.”

Hunter continues:

By the beginning of December he had space reserved, mailing lists set up, Stanford's financial folks on board, and the call for papers out—everything went surprisingly well, and in the end, very few people knew that we had done such drastic mid-course corrections.

Chris Rawlings recalls how hectic it was organizing the third conference at Cambridge:

[At the second conference] we basically said, “Well, we should organize one in the UK. It shouldn't just be a US thing.” So we put our hand out and said, “Yeah, OK, we'll do the next one.”…it was done on a shoestring. That was the real challenge, doing it without any huge support from your organization, who didn't really know why you were doing it and were worried that you were going to make a financial loss, and therefore have to underwrite it. It was just all the practical things that got in the way, and underestimating the amount of work that was needed.

The international character of ISMB has been important from the outset. In the preface to the proceedings of the first ISMB, the full name of the gathering is stated as the “First International Conference on Intelligent Systems for Molecular Biology”. The preface continues:

The word “international” in the title reflects the observation that outstanding work in this field takes place in many countries around the world. Not only was the program committee drawn from Europe, North America, and Asia, but a gratifying fraction of the submissions were as well.

The commitment to an international conference has only grown stronger over the years. Thirteen of the conference's 20 years have been held outside of the US. In recent years, the rotation of conference venues has formalized, with a European country hosting every other year, and North America hosting on intervening years. Also, since 2004, ISMB has been held jointly with the European Conference on Computational Biology (ECCB) during each European meeting.

Beyond the organizational challenges of the early conferences, the format of ISMB was an issue as well. Peter Karp recalls:

One thing about our field is we were really starting a new science, and there were a lot of decisions that we got to make about how to shape the field in a number of different ways. For example, how would conferences be run. Some of the choices we were making were, for example, would we have refereed proceedings from our conferences or not? We decided, yes, we would do that. That's how computer science works, but it's not how biology works. The way biology works is most of the speakers are usually invited. That's not how we did it because we kind of wanted to give everyone a chance with setting up their ideas and have reviewers decide what seemed to be the best ideas. That seemed to be a more open way to run things.

Initially, the refereed papers were presented in a single track. It wasn't until the 2004 meeting in Glascow, Scotland, that the conference moved to parallel tracks for paper presentations. Rawlings:

That was quite controversial in the history of the conference. There were a lot of people who wanted to keep it more strongly in the AI intelligent systems model and have a meeting where everybody would go to everything. But it just grew too big. We just couldn't…. The other thing that was unique amongst conferences in the early days was that we followed a tradition from computer science conferences of having the tutorials before the meetings.

By 2007, ISMB had expanded so dramatically that each attendee could choose from among 23 pre-conference offerings, including special interest group meetings, tutorials, and a student symposium. The main conference had expanded to more than 150 talks spread over eight parallel tracks.

Altman remarks on growth and specialization of the conference as well:

I could go to ISMB '93 or '94, and I could sit in on every talk and understand every word of every talk because it was a new field. There wasn't differentiation yet. We all were generalists. Then you fast-forward 20 years, and we now have areas of specialty and expertise. And I can go to a talk where I may understand the rough idea, but I haven't worked in that field, and a lot of the basics are not immediately familiar to me…it's just a very natural evolution of any [new] field. But there's always a certain bit of sadness, because you yearn for the days when you could sit in a room and understand every talk. And right now that's getting harder and harder.

Alfonso Valencia identifies ISMB meetings that marked important milestones:

The [1995] conference in Cambridge was the year where the conference changed from a computer science-based conference to a point where everyone realized that, if you want to make progress, there has to be more focus in biology. So this is where there was a significant change in the spirit and the orientation of the conference.

The [1997] one in Greece was important because it was the first time we realized that we needed a professional [society]…and where we founded ISCB. [It established the conference as] part of our professional activities, as *the* conference for bioinformaticians and computational biologists.

The [2004] one in Glasgow was the one where we realized we were really big because, for the first time, the numbers were over 2,000 people. And it was the first one where the balance between Europe and the States became an important part of the conference. It was here that we established the rules and the ways and the spirit of collaboration between the Americans and the Europeans.

Valencia further emphasizes ISMB's substantial contribution to forming the identity of the computational biology profession. Searls agrees, summing up the road ISMB has traveled:

There were no grizzled veterans back in the early days. There were no gray eminences, no old-timers. It's not a question of age…. It's a field that started out without any infrastructure, without any standard curricula, without any good journals, and without a meeting series like ISMB. That's something that's all been addressed, one by one.

The field has developed an infrastructure, for lack of a better word, which makes it an authentic scientific field, and now you do have a sense that there are standard curricula, that you know what you need to learn in order to be educated in this field now.

**Figure 1:**
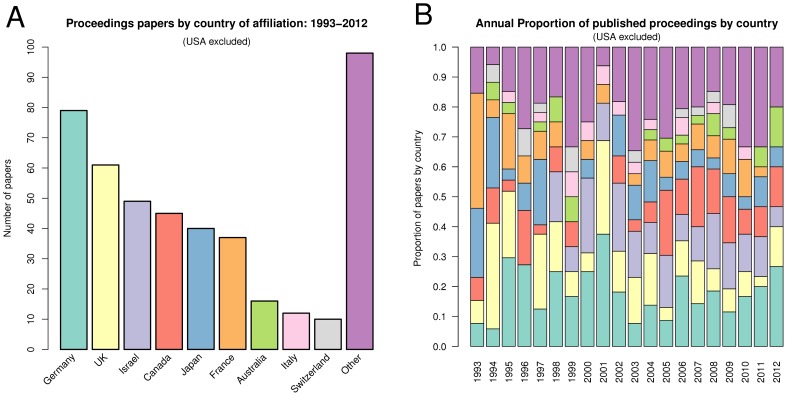
Proceedings papers by country. Shown are countries from which at least ten papers have been published across the 20 ISMB meetings. (A) The total numbers of papers originating from each country across the 20 ISMB meetings. The USA is not shown (472 papers). The “Other” category is the aggregate of countries having fewer than ten papers: Denmark (9), Finland (8), Russia (8), Belgium (7), China (7), Netherlands (7), Sweden (7), South Korea (6), Austria (5), Mexico (4), Spain (4), Brazil (3), Ireland (3), Singapore (3), Taiwan (3), India (2), Latvia (2), New Zealand (2), Norway (2), Poland (2), Chile (1), Herzegovina (1), Hong Kong (1), and Philippines (1). (B) The proportion of publications from each country for each year of the conference. The USA is not calculated into the proportion.

The proceedings papers from the first ISMB meeting are affiliated with eight different countries. One sign of ISMB's maturity is that 34 country affiliations have now appeared in the ISMB proceedings over the last 20 years (see Figure 1). The conference's growth is also evident in the increasingly competitive acceptance rate (see Table 1).

**Table 1:** ISMB Paper acceptance rate.

Year	Papers Accepted	Papers Submitted	Acceptance Rate
1993	52	69	75%
1994	47	86	55%
1995	48	88	55%
1996	27	65	42%
1997^a^	54	85	64%
1998	25	92	27%
1999	34	91	37%
2000	41	141	29%
2001	38	180	21%
2002	42	207	20%
2003^a^	48	341	14%
2004^a^	67	492	14%
2005	56	428	13%
2006	67	404	17%
2007	66	417	16%
2008	48	287	17%
2009	46	242	19%
2010	48	235	20%
2011	48	258	19%
2012	35	268	13%

Rate of papers accepted into ISMB proceedings.

a Calculation includes short papers.

## The Conference Title

Perhaps the most enigmatic feature of the largest international conference in computational biology is the name of the conference itself: Intelligent Systems for Molecular Biology. The conference owes its pedigree to AI. The database from which Hunter drew the invitee list for the November 1991 pre-ISMB workshop was of AI researchers with an interest in molecular biology. Hunter:

We spent a lot of time until at least '96, arguing about the name. “Intelligent Systems for Molecular Biology” came about because we thought at the time that it was going to be artificial intelligence for the software piece and laboratory robotics was going to be a big piece, instrumentation. Intelligent systems was supposed to include robotics.

We had all these grand ideas—it was sort of [based on] how the genome project was going to advance, there would be both instruments and computer science. So that's where the Intelligent Systems piece came from—it was supposed to be not just AI, but also robotics.

Searls also recalls the difficulties in settling on a title for the conference:

We talked about that quite a while. Part of it was, we wanted to establish this community that was interested in symbolic approaches to biology. We didn't want to name it “AI” because our focus wasn't on machine intelligence. It was on actual applications.

We thought “Intelligent Systems” would be a general enough description of the kinds of methodologies that we were interested in, that would keep the focus on biology. In fact, even the choice of the word Intelligent Systems “for” Molecular Biology was chosen as opposed to “and” molecular biology, because we didn't want this to seem like, “This is a computer science meeting and a biology meeting.” It's computer science in service of biology.

We thought long and hard about the title. We wanted to keep it associated with this [developing] community, which in large part grew out of the AI community at the time. But at the same time we wanted the focus to be on the biology.

Finding a term related to “artificial intelligence” that had an appropriate scope was only part of the difficulty in naming the conference. As Searls touched on, there was also difficulty properly balancing the computational versus biological components of the title. Altman also notes this balance:

Although other people, I think, rankled a little bit about “Intelligent Systems for Molecular Biology”, I thought it was brilliant. I still think it's brilliant because I think it has this implication that we're not just writing utilities to analyze biological data. We're going for something much bigger than that, which is innovative, even discovery-oriented software that can either make hypotheses or prove hypotheses….

I've been very strong to stay away from the idea of computation as service in biology because I think it demeans the profession, and there are too many biologists who would be happy to have informatics people subjugated as service types. So I would be very sensitive to that.

Richard Lathrop provides a detailed deconstruction of the title:

There are several parts to that, the first of which is, why “intelligent systems”, the second is, “why biology”, and then the third is, why are they connected by a “for”?

I think the reason for “intelligent systems” is because intelligent systems are the class of computational tools best able to handle complexity with grace. And biology is just absolutely full of complexity. It is the robustness and the grace with which intelligent systems handle that complexity that makes them especially well-suited.

Then there is the why the “molecular biology”? And by now, we construe that to be generally biology in medicine. The answer is that they're generating vast amounts of data, and locked in that data are, on the one hand, the answers to questions that people have wondered about for millennia, and on the other hand, a lot of dramatic cures to diseases, improved agricultural products, a number of things that will directly benefit life today.

And then the question is why the “for”? And we really wanted this to be, in a sense, in the service of molecular biology, in the sense that that's where the content is…. Molecular biology has the content, that's the original source of the knowledge. It's the source of the data that contains the knowledge. And so the “for” is there in the sense that it wants to be intelligent systems for the content and the knowledge that's encoded in the molecular biology, rather than trying to make them equal or the molecular biology handmaiden to the computation. Definitely the computation is a handmaiden to the molecular biology.

One of the more interesting issues in the responses above is that of service. An initial interpretation suggests that there is sharp disagreement between whether bioinformatics (or computational biology) is properly characterized as computer science “in the service of” biology. However, there is a subtle distinction being made between the techniques and the roles of the profession. That is, computer science *methods and algorithms* in the service of biological *data*, versus computer *scientists* in the service of *biologists*.

## On the Dichotomy between Computer Scientists and Biologists

The ISMB meetings are interdisciplinary in nature, and the relationship between biologists and computational scientists has evolved over the last 20 years. Lathrop has seen a dramatic change:

When I first began this, there was a very common response, especially among senior biologists, that: “computational biology is just a faster way to do theoretical biology, and we all know that theoretical biology doesn't work. And so computational biology is just a way to do something that doesn't work even faster.”

And today, I think, almost all biologists, especially the ones that are involved in any of the modern data generation technologies, recognize that the future of their discipline is in computation, because of its efficiency of turning data into knowledge, which is what they want in the first place when they do a genome-wide association scan or a deep sequencing project or X-ray crystallographic analysis, or a gene array experiment. It's literally impossible for them to sit down with a paper and pencil and go through all of those data points.

And [even] if they could, they would miss most of the sophistication of the statistical and other methods that have been coded into the algorithms. And so I think one of the big sea changes is that the biologists have become much more enthusiastic about the idea that computation benefits biology.

Milton Corn also recognizes the changed relationship, but is more cautionary about its continuing evolution:

The marriage of biology and computational stuff is still an uneasy one. The biologists now accept the need for computation, but I think they tend to think of the people who do this, the computer scientists, the engineers, mathematicians, as people who are very useful for producing tools that the biologists can use.

And the computer scientists, engineers, etc., sometimes are quite naive about the complexity of biologic problems. When they're first asked to give a hand to a biologic problem, they think, “Oh, there's nothing to this. We have hundreds of solutions already sitting here, and all we have to do is to apply them to the genome or the systems modeling.” It's a big shock to them when it turns out that doesn't work.

What we need more of, in order to get a good partnership between computational people and the biologists, is a little more respect on each side, for the complexities and needs of the other.

Shavlik saw this dichotomy during the grant-review process as well:

When I was on a review panel for NIH for four years it would drive me crazy when other reviewers would say (and maybe I'm caricaturing a bit) “[new algorithms] should just be funded by NSF, we only want to fund when the computation's all worked out.”

Altman defines when the bioinformatics role is more appropriately viewed as a service position:

In some ways, the world is our oyster because there is so much biological data that it has become obvious to everybody that you need to have a strong informatics component to many research projects.

There is still [a lack of] clarity sometimes about whether the informatics person should be a co-equal colleague of the biologist or should be a service. And of course there are some service functions. Are you using off-the-shelf software that's already been invented and you're just turning the crank? That's service and appropriately not co-equal. But if there are novel methods required, then it's not service: you're creating new methods as a colleague in a scientific enterprise.

## Twenty Years of ISMB: Maturation of the Discipline

When the interviewees discussed the changed landscape over the last 20 years, the explosion of data and the trend toward methodologies that integrate different types of data were common themes. Lathrop and Hunter identify how researchers' dispositions have changed along with the data. Lathrop:

It's been dramatic. The breadth and sophistication of the entire enterprise has just been astounding. When I first started this, GenBank and the Protein Data Bank and the DNA Database of Japan did not even cross-index each other's loci IDs….

When I started, nobody had even broached the idea of sequencing the human genome. It was just too gargantuan and mammoth a task, and was considered almost heresy in its early days. Many biologists were rabidly opposed to it, because of how much money it would take away from small science and individual biologists doing work in their own labs.

Hunter:

It used to be the case that you could really separate people out by the sort of data they worked on. There were people who looked at crystallographic protein structures, and there were people who looked at amino acid sequences and did primary sequencing analysis and there wasn't much overlap between them. There wasn't that much overlap between the people who looked at nucleotide sequences and the people who looked at amino acid sequences.

Everybody had a problem that was tailored to one particular, I wouldn't say instrument exactly, but one particular data generation modality. That is increasingly less true…increasingly these days the idea is to try to merge multiple sources of information. The sources of information keep growing and there are new ones all the time but the trend [is] towards trying to use more than one kind at a time.

Hunter also credits instruments as a driving force in the field:

Computational biology seems to go in a spiral rather than a circle where we keep revisiting topics that we think were solved. In 2001 we thought that sequence analysis was a solved problem and microarray analysis was where the action was. In 2012 it's the other way around.

We are driven largely by the instruments and so now sequencing, which we really did think was a solved problem, is really interesting again. In part because short reads are different than the 500 base pair reads that we got out of previous technology, but also because it's now gotten so cheap we can do all kinds of things that would have been crazy to do 10 years ago.

Karp reflects on how the models have changed:

The progress made by the field has been really amazing…the ability to go from a genome, a list of As, Cs, Gs, and Ts, to a mathematical model of the metabolic network of the organism.

There's a whole series for computational processing steps that can be used to infer a…steady state mathematical model from its genome sequence….it really reflects 20 years of progress in many different areas of bioinformatics that have all been tied together…. If you asked me 20 years ago, would the field have been able to do that, I would say “no way”.

**Figure 2:**
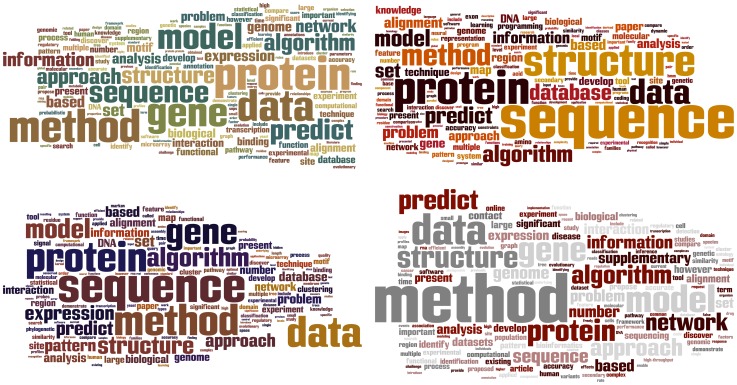
Proceedings titles and abstracts. Which word cloud reflects each five-year time period of ISMB? Each word cloud represents five contiguous years of conference titles and abstracts: 1993–1997, 1998–2002, 2003–2007, 2008–2012. The order of the clouds has been scrambled and can be found in the Acknowledgments. H/t, Jude Shavlik.

The changes can also be seen in the word frequencies of the published proceedings. In Figure 2, the proceedings titles and abstracts are presented as four word clouds. Each word cloud represents an out-of-order 5-year period of ISMB—what is the correct temporal ordering?

## Emerging Trends and the Future

As the interviewees turn their attention to the present and future, the volume of data generally and next generation sequencing specifically is mentioned by several. Searls:

I think everybody's excited about next-gen sequencing. Well, they're either excited about it or they're scared to death of it.

To put a positive spin on it, I think the fact is that it is going to be producing such a huge volume of data, but in so many different aspects of biology. I think that's got to be the most interesting thing on the horizon now, is how do we simultaneously deal with volume and diversity?

The fact that we're now able to start to deal with—I'll pick epigenetic data in particular as an exciting area, another one would be cancer genomics—where the biology has matured to a point where the data generation is now at such a level of detail, and so massive in scale, that the computational challenges are just vast and out-of-this-world complex.

That's the sort of environment where you need to have new computational paradigms…. There's going to be new, emergent computational approaches that are going to be necessary for dealing with the explosion of data at the moment.

Altman zeroes in on sequencing algorithms:

I think it caught us all by surprise, the rate at which next-generation sequencing hit the world and created a demand for informatics skills that had a little bit gone out of style. So people who did sequence assembly, sequence mapping, we thought we had that under control because the throughput was such that we could handle it with existing algorithms. But when next generation sequencing came to the scene, it immediately showed us how inadequate a lot of our algorithms were…. That's not even a trend. We're sitting in the middle of it right now.

Altman, along with Valencia, also note current translational biomedical trends. Altman:

The other trend is the interest in applying bioinformatics and computational biology to translational medicine and to the clinic. I, myself, have gotten caught up in this. I'm an MD, so this is not a huge surprise. But for many years, even though I was a physician, my work was very basic and did not have any translational component.

Valencia:

Biology is getting closer to biomedicine so we have all of these talks of how to analyze genomes in a biomedical context. And that is going to be very influential in the future. Of course, we have the influence of sequencing that is related to that, the sequencing of genes. We need a new method to analyze the information. That is very influential and fascinating and growing really fast. Image analysis is still small but is going to be very important.

Karp identifies some expectations:

What's striking to me now is how little we can learn from an individual's genome right now…we can sequence somebody's genome, and we've learned virtually nothing with any reasonable amount of certainty…I sure hope that in 10 or 20 years, we can do a lot more with a personal genome…predict not just that they're five percent more likely to get some disease, but that they're 90 percent more likely to get one or more diseases. And the treatment that will help that individual. I also hope we'd be able to create mathematical models of communities of bacteria that exchange nutrients and interact with one another.

## Contributions to Other Fields

Bioinformatics draws from many areas of mathematics, statistics, and computer science in its quest for biological illumination. But what about the reverse? What contributions has ISMB and bioinformatics generally made to other areas of research? Altman identifies big data:

I think that in a funny way, bioinformatics led the big data craze…. In 1998, way before the Google clickstream was significant, we were looking at what was at the time very large data sets and trying to figure out how to get them under control …. Some of the basic things like clustering and heat maps of clusters, where you take a big matrix of data and then you do a clustering of the rows and the columns, I'm pretty sure that the…visualization of that and the metaphor for display goes back to the 1998 paper by Mike Eisen on clustering of yeast microarray data.

Searls notes that the complexity endemic to biological data is an important contributor:

If I had to pick one thing that biology was giving back to computer science, it's simply the driving examples and the fact that there's so much biology that it really exercises everything that you'd want to work on, in at least certain branches of computer science.

In the early days of algorithms, people who did string algorithms, for example, at first they were interested in biological applications mainly as just driving examples for developing new string algorithms, proving some theorems. Biology has provided some nice examples for the introductions of their papers, then they would go off and prove theorems….

I think those days are gone. If you look at typical engineering departments in my alma mater at MIT, the engineering departments have actually stated openly that biomedical applications are going to be their main driver. Not just of electrical engineering and computer science, but other departments as well, mechanical engineering and so forth.

Shavlik picks up on this as well:

[Algorithm families] might have started elsewhere but they often got a lot of visibility by showing special cases, improvements, and good results on challenging biological problems.

The ISMB community was ahead of maybe other parts of computer science in appreciating the role of machine learning and lots of data, shared data sets on the Web–GenBank, Protein Data Bank, and all those resources that molecular biologists widely shared were very valuable to computational people.

Some that I see having a big impact in ISMB are things like Hidden Markov Models and Support Vector Machines, and Bayesian networks and other graphical models, probablistic models. Those all had some beginnings in other things. I think the…challenging and realistic…problems that computational biology, computational medicine bring up, show those algorithms being really useful on important problems.

The algorithms get changed due to the data sets they're tested on. A whole family of algorithms, the broad framework might have been defined before such as neural networks or Bayesian networks but some of the exact details really get pushed because of the test bed you're working on.

Rawlings and Shavlik both note the iterative nature of technology adoption. Rawlings:

An area that's small suddenly booms as a technology, and then gets fed back. Neural networks probably was one area with relatively small activity that was picked up as a technology in the ISMB community. It became very much bigger as a field of people applying neural networks, and machine learning too. It's still a very strong tradition in the machine learning community that the molecular biology domain is seen as one of those crucial domains that help grow that community. They still use data sets from [molecular biology] in their test examples that are used by people working in machine learning for its own sake.

Shavlik:

I think there's a tendency to think computer science people develop the algorithms and then some other people just apply them blindly and find biological results. But there's a feedback loop where the problem one works on guides what weaknesses you see in existing algorithms and what weaknesses a new technique tries to overcome. It's more of a loop, a synergistic feedback, rather than a linear thing.

## The Human Element

Beyond the physical location of the conference, the people are the essence of ISMB's international character. Typically, 60% of the attendees come from the continent of the meeting's location. The men and women comprising the ISMB organizing committees and invited keynote speakers have remarkably diverse geographic origins as well. Many of the interviewees characterized this ISMB community as a gathering of colleagues and friends. Hunter and Karp offer excellent summaries. Hunter:

In my mind, the most important thing about ISMB is community, is meeting people that you don't otherwise have an opportunity to meet, to really talk to them face-to-face. Chance encounters are important. Updating your community on stuff you've been up to. For a graduate student, you don't really know anybody and so you're meeting both your peers, other graduate students, and then the more successful people in the field.

Karp:

One of the things I like about this field is the people are really great. They're very interesting people, very smart people, people who enjoy life in a lot of ways.

This was a recurring theme among the interviewees. They talked of lifelong friendships formed out of working through ISMB's early challenges together, and of the enjoyment of spending time with colleagues between paper sessions and during ISMB's social events. The tone ISMB sets for the computational biology community has undobutedly contributed to the profession being an avocation for so many. And as sexy as Hollywood cinema may be, its AI community is poorer for it.

## The Personalities

The people interviewed were all instrumental in the formation and early years of ISMB, and many are still active in computational biology and ISMB today. However, many other folks who were not interviewed deserve equal credit for their contributions to the formation and continued success of ISMB. Table 2 lists the people who participated in the November 1991 pre-ISMB workshop. The conference chairs for each year of the conference can be found in Table 3.

**Table 2:** Pre-ISMB workshop attendees (November 1991).

Amos Bairoch	Lawrence Hunter
Ann Barber	Peter Karp
David Benton	Toni Kazic
Douglas Brutlag	Richard Lathrop
Christian Burks	Hwa Lim
Su-Shing Chen	Michael Mavrovouniotis
YT Chien	George Michaels
Dominic Clark	Harold Morowitz
Peter Clepper	Mick Noordewier
Milton Corn	Scott Presnell
Charles Coutler	Chris Rawlings
Philip Curtiss	David Searls
Dan Davison	Jude Shavlik
Chris Fields	David States
Robert Futrelle	Martin Stodolsky
Michael Gribskov	Gary Stormo
Caroline Holloway	John Wooley
Tim Hunkapiller	Maria Zemankova

Listed are those who participated in the November 1991 pre-ISMB workshop.

**Table 3:** ISMB conference chairs.

Chair	Year	Chair	Year
Lawrence Hunter	1993	Goran Neshich	2006
David Searls	1993	Ana Tereza Ribeiro de Vasconcelos	2006
Jude Shavlik	1993	Thomas Lengauer	2007
Russ Altman	1994	Burkhard Rost	2007
Chris Rawlings	1995	Peter Schuster	2007^a^
Pankaj Agarwal	1996	Thomas Hudson	2008^a^
David States	1996	Michal Linial	2008
Christos Ouzounis	1997	Jill Mesirov	2008
Janice Glasgow	1998	Burkhard Rost	2008
Thomas Lengauer	1999	Gunnar von Heijne	2009^a^
Philip Bourne	2000	Eugene Myers	2009
Michael Gribskov	2000	Marie-France Sagot	2009
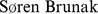	2001	Michal Linial	2010
Anders Krogh	2001	Jill Mesirov	2010
David Wishart	2002	Olga Troyanskaya	2010
Eugene Myers	2003	Michal Linial	2011
Mark Ragan	2003	Burkhard Rost	2011
David Gilbert	2004	Peter Schuster	2011^a^
Janet Thornton	2004	Kurt Zatloukal	2011^a^
Brian Athey	2005	Sydney Brenner	2012^a^
David States	2005	Terry Gaasterland	2012
		Richard Lathrop	2012
		Burkhard Rost	2012

The conference chairs and co-chairs for each of ISMB's 20 years.

a Honorary.

### Russ Altman

Russ Altman was an organizer of the second ISMB meeting at Stanford University in Palo Alto, California, and the third ISMB meeting at Robinson College in Cambridge, UK. Today Russ Altman is Professor of Bioengineering, Genetics, and Medicine at Stanford University.

### Milton Corn

When Hunter started work at NLM and during the time ISMB was formed, Milton Corn ran the grants program at the National Library of Medicine. Today Milton Corn is Deputy Director for Research and Education at the National Library of Medicine, and continues to be Hunter's program officer today.

### Lawrence Hunter

Lawrence Hunter was an organizer of the first ISMB meeting at the National Library of Medicine in Bethesda, Maryland, the third ISMB meeting at Robinson College in Cambridge, UK, and the fourth ISMB meeting in St. Louis, Missouri. Today Lawrence Hunter is Professor of Pharmacology and Computer Science at the University of Colorado.

### Peter Karp

Peter Karp was an organizer of the second ISMB meeting at Stanford University in Palo Alto, California, and the fifth ISMB meeting in Halkidiki, Greece. Today Peter Karp is Director of the Bioinformatics Research Group in the Artificial Intelligence Center at SRI International.

### Richard Lathrop

Richard Lathrop was an organizer of the second ISMB meeting at Stanford University in Palo Alto, California, and the sixth ISMB meeting in Montréal, Canada. Today Richard Lathrop is Professor of Computer Science at the University of California, Irvine.

### Chris Rawlings

Chris Rawlings was an organizer of the third ISMB meeting at Robinson College in Cambridge, UK. Today Chris heads the Department of Computational and Systems Biology at Rothamsted Research, UK, and is a visiting Professor of Bioinformatics at Imperial College, London.

### David Searls

David Searls was an organizer of both the first ISMB meeting at the National Library of Medicine in Bethesda, Maryland and the second ISMB meeting at Stanford University in Palo Alto, California. Today David Searls is an independent biotechnology professional and was formerly Senior Vice President of Computational Biology at GlaxoSmithKline.

### Jude Shavlik

Jude Shavlik was an organizer of the first ISMB meeting at the National Library of Medicine in Bethesda, Maryland. Today Jude Shavlik is Professor of Computer Sciences and Biostatistics & Medical Informatics at the University of Wisconsin-Madison.

### Alfonso Valencia

Alfonso Valencia was an organizer of the fifth ISMB meeting in Halkidiki, Greece, and the 16th ISMB meeting in Toronto, Canada. Today Alfonso Valencia is the Director of the Spanish National Bioinformatics Institute.

